# Applying an Integrated System of Cloud Management and Wireless Sensing Network to Green Smart Environments—Green Energy Monitoring on Campus

**DOI:** 10.3390/s22176521

**Published:** 2022-08-29

**Authors:** Kuo-Hsiung Tseng, Meng-Yun Chung, Li-Hsien Chen, Ming-Yi Wei

**Affiliations:** 1 Department of Electrical Engineering, National Taipei University of Technology, Taipei 10608, Taiwan; 2 Department of Civil Engineering, National Taipei University of Technology, Taipei 10608, Taiwan

**Keywords:** cloud management, composite green roof, green information system, environmental greening benefits

## Abstract

With increasing urbanization, the application of Internet of things (IoT) technology to city governance has become a trend in architecture, transportation, and healthcare management, making IoT applicable in various domains. This study used IoT to inspect green construction and adopted a front-end sensing system, middle-end wireless transmission, and a back-end multifunctional system structure with cloud management. It integrated civil and electrical engineering to develop environmental monitoring technology and proposed a management information system for the implementation of green engineering. This study collected physical “measurements” of the greening environment on a campus. Ambient temperature and humidity were analyzed to explore the greening and energy-saving benefits of a green roof, a pervious road, and a photovoltaic roof. When the ambient temperature was below 25 °C, the solar panels had an insulation effect on the roof of the building during both 4:00–5:00 and 12:00–13:00, with an optimal insulation effect of 2.45 °C. When the ambient temperature was above 25 °C, the panels had a cooling effect on the roof of the building, whether during 4:00–5:00 or 12:00–13:00, with an optimal cooling effect of 5.77 °C. During the lower temperature period (4:00–5:00), the ecological terrace had an insulation effect on the space beneath, with an effect of approximately 1–3 °C and a mean insulation of 1.95 °C. During the higher temperature period (12:00–13:00), it presented a cooling effect on the space beneath, with an effect of approximately 0.5–9 °C and a mean cooling temperature of 5.16 °C. The cooling effect of the three greening areas on air and ground temperature decreased in the following order: pervious road > photovoltaic roof > ecological terrace.

## 1. Introduction

The global environment is burdened by human development. With population growth, environmental pollution, and climate change, many sectors have begun to improve their living environment to achieve the goal of sustainable development. Currently, environmental engineering mostly applies the long-term monitoring of environmental pollution and employs greening techniques; however, it rarely includes the “benefits of greening engineering” among the items it inspects. In the early 19th century, the global population surged rapidly, and the Industrial Revolution flourished when social and scientific conditions were met. Enormous changes in the environment, as well as the greenhouse effect, have occurred because of large-scale human activity and development and excessive greenhouse gas emissions, which have led to global warming, climate change, and changes in other environmental factors [1,2]. Because of the major problem mentioned previously, a method to protect the environment was proposed recently. The Vertical Greening Modular System (VGMS) is an increasingly popular building envelope solution designed to enhance the aesthetic quality of new and existing façades while achieving energy-efficient performance. The VGMS was designed and tested in a research project in Turin (northern Italy). The VGMS achieves a 40% reduction in the equivalent thermal transmittance measurement, significantly affecting the amount of energy passing through the exterior wall during warm seasons. The outdoor surface temperature of the wall was reduced by 23 °C in summer, which had a positive impact on outdoor comfort and urban heat island mitigation [3]. Another research target was focused on pavement watering. A field study conducted at the Louvre in Paris in the summer of 2013 showed that watering from the pavement lowered the temperature in the afternoon by 5.9 °C [4]. A study on a new PV panel thermal model at the roof laboratory of the Czech University of Life Sciences analyzed the differences between freestanding and roof-integrated configurations in different locations (cold, medium, warm, hot). The result showed that the panel can reduce annual power generation by 3–4% (cold, mild, warm) and by more than 5% in hot climates [5].

This study used relevant technology to assist with urban governance, green engineering techniques, and renewable energy to develop urban construction. Furthermore, Internet of things (IoT) technology was incorporated to improve urban environments and energy-saving problems for cities, where clean energy and environmentally friendly approaches were utilized to facilitate clean urban environments and sustainable development. Based on two elements, environmental engineering and architecture energy saving, an examination of energy saving and the greening benefits of green construction in civil engineering was implemented. The advantages of photovoltaic reusable energy in electrical engineering for cooling and power-saving in buildings were also examined. The exploration of the interaction between greening and energy saving and urban environments can provide city governments with valid information, strengthen the development of urban green environments, and enhance the growth and promotion of sustainable cities [6,7,8]. Based on the concept of smart cities under the application of Internet of things technology, this paper set up a wireless environmental monitoring and management system in the campus environments of urbanized cities to monitor and quantify the benefits of greening projects to the campus environment over a long period [9,10,11]. This research analyzed three different cases. The subjects were a green roof, a pervious road, and a photovoltaic roof. The quantitative results can be employed in estimating and calculating energy saving/consumption or carbon emissions and serve as a reference for urban governance. The monitoring and analysis of the environmental benefits of the actual greening project site in this study were as follows:

1. Collected green roofs, permeable pavements, and solar photovoltaic system benefit evaluations for the application representatives of the three indicators of “greening”, “base water retention”, and “daily energy saving” in the green building evaluation system.
2. Integrated sensor technology and cloud monitoring to build a campus green information system.
3. Analyzed the impact of environmental factors on the power generation efficiency of the photovoltaic system, the effect of roof greening and photovoltaics on the cooling effect of building shading, and the environmental greening benefits of permeable pavement.

## 2. Materials and Methods

### 2.1. System Architecture

Smart environments have measures for strengthened environmental monitoring. A subdevelopment project for smart cities is gathering environmental information through ICT, inspecting the environment using data analysis methods, and formulating environmental management improvement plans to address environmental problems and enhance urban residential environment safety and quality [12]. Rapid, massive industrialization and urbanization have placed heavy burdens on the environment in Taiwan; therefore, environmental management strategies should be comprehensive, with enhanced environmental conservation concepts. The Industrial Economics & Knowledge Center (IEK) Net of the Industrial Technology Research Institute (2016) specified three concerns regarding domestic smart environment management:

1. Lack of environmental data due to markedly few environmental quality sensors;
2. Lack of measuring points due to the high cost of environmental quality sensors;
3. Lack of overall planning due to insufficient experience in solving environmental problems through big data analyses.

In addition to these concerns, difficulties regarding environmental monitoring, including the expiration of sensors, the harshness of the monitored environment, and problems of transmission and electricity because of remoteness occur. Thus, the establishment of environmental monitoring is extremely challenging. However, effective applications of environmental monitoring data are expected to reduce ecological and environmental damage, support the establishment of effective management strategies, and prevent artificial damage and natural disasters [13,14]. 

**Figure 1:**
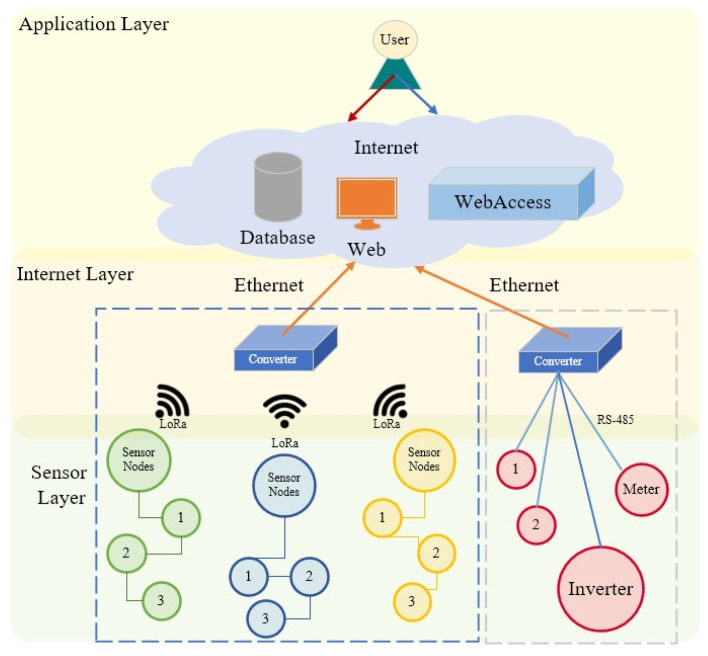
Structure of the campus green information system (the 1 to 3 stands for different sensor nodes).

This study constructed a green information system using IoT technology, as depicted in Figure 1. The monitoring data of the perception layer were relayed to data convertors through a wireless network in the network layer and forwarded to servers in a physical network. A cloud management system was then constructed to facilitate user management.

### 2.2. Equipment

#### 2.2.1. Sensors in the Perception Layer

SHT-10 temperature and humidity sensors were established in the perception layer to monitor the benefits generated by environmental greening. The sensors were equipped with both sensor elements and signal processing functions, and every sensor was individually calibrated in a precise humidity cabinet. They provided fully calibrated digital output and measured relative humidity and temperature using capacitive sensor elements and bandgap sensors, respectively. CMOSens technology was adopted to ensure favorable reliability and long-term stability. 

#### 2.2.2. Long-Range Wireless Data Transmission Technology

The study primarily monitored the benefits of environmental greening, which is a type of environmental monitoring in smart cities [15]. Considering the development and challenges of smart cities and the problems they are likely to encounter during environmental monitoring, a long-range (LoRa) low-power wireless data transmission technology was adopted as the transmission interface for transmission and communication in the monitoring system. The LoRa technology was presented by the Semtech Company in August 2013 and is categorized as a type of low-power, wide-area network (LPWAN) [16]. Wireless communication transmission was originally based on Wi-Fi, 3G/4G, and ZigBee; LPWAN technology was designed to resolve the problem of transmitting few data over a long distance. LoRa has a transmission distance range of 1–15 km and is relatively power-saving. LoRa, with 200 pieces of transmission data per day, is a suitable communication transmission interface, particularly for industrial IoT, smart cities, and quality agriculture, though it is unfeasible for transmitting large files, audio, or video. Communication techniques have advantages and disadvantages. The AS32-TTL-100 LoRa transmission model was selected for data transmission in the monitoring system because all sensing positions in the monitored environments were established outdoors and the number of test points was likely to be expanded. 

#### 2.2.3. Advantech WebAccess

The application layer was developed using Advantech WebAccess (hereafter referred to as WebAccess) software. WebAccess is a human–machine interface, supervisory control, and data acquisition system developed for IoT technology. It can be used for animated graphic displays, real-time data, control, trends, reporting crimes, and keeping daily journals, and these functions are available on standard web browsers. This study employed a campus environment green information system and WebAccess graphic software for data extraction, computing, and management. WebAccess professional edition was used because it can manage 1500 monitoring points and up to 255 devices; in addition, the professional edition does not limit the number of webpage users, which facilitates multi-person connection, browsing, and management.

### 2.3. Greening of the Monitored Area and the Green Information System

This study adopted a campus reusable energy system for environmental monitoring at three green engineering sites, namely, a photovoltaic roof, an ecological terrace, and a pervious road. The monitored area and placement of sensors were discussed in [17,18,19,20]. To understand the benefits of green engineering for the cooling of the environment, the three greening positions were introduced and monitored for temperature and humidity both in the air and on the ground. 

#### 2.3.1. Photovoltaic Roof

**Figure 2:**
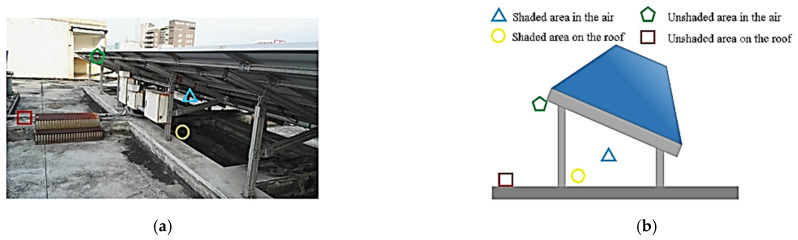
(**a**) Photograph and (**b**) schematic of the sensing positions on the photovoltaic roof.

A photovoltaic system environment was constructed on the roof of a campus building. Bother fixed- and single-axis photovoltaic systems were installed with a total capacity of 70.38 kWp, and monitoring equipment was set up. To monitor the influence of solar panels on the cooling effect on the top floor and that of ambient temperatures on the power generation of the solar modules, this study examined the sensing approaches of the solar panels with regard to ambient temperatures and the shading effect [21,22]. The fixed photovoltaic system and areas of the roof were selected as sensing areas, and temperature and humidity sensors were placed in four locations: in the air under the solar panels, on the ground in a shaded area, in the air without shade, and on the roof without shade [23,24,25]. The sensing positions are depicted in Table 1 and Figure 2. 

#### 2.3.2. Ecological Terrace

**Figure 3:**
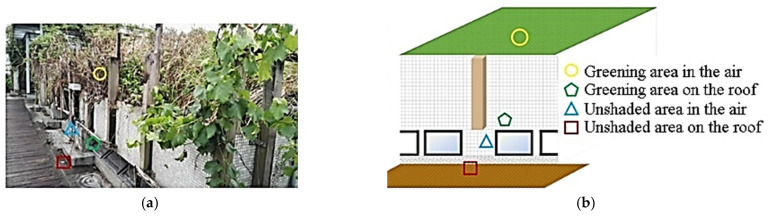
(**a**) Photograph and (**b**) schematic of the sensing positions on the ecological terrace.

An ecological terrace, a type of green roof, on a campus building was selected for examination [26,27]. The building was built according to the campus eco-environment design plan to provide biological habitats and restore urban green spaces. The plants on the terrace absorb CO_2_ and have a shading effect on the building; the floor area is approximately 80 m^2^. This study selected the greening area and an unshaded area on the roof as sensing areas and placed temperature and humidity sensors in four locations: in the air within the greening area, on the roof in the shade of the greening area, in the air without shade, and on the roof without shade. A sensor was also placed in a classroom beneath the ecological terrace. The sensing positions at the ecological terrace were as described in Table 2 and Figure 3.

#### 2.3.3. Pervious Road

**Figure 4:**
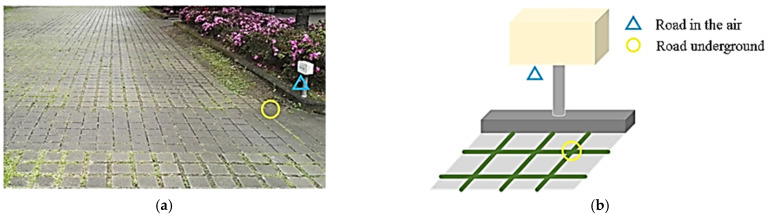
The sensing positions on the pervious road: (**a**) photograph and (**b**) schematic.

Three types of water-pervious roads were discovered at the campus, namely, pervious concrete, interlocking block, and grass block paver roads, representing the three major approaches to pervious roads currently used (i.e., material, splicing, and structural brick). This study selected the grass paver road (Figure 4). The total area of the pavement was approximately 800 m^2^. According to monitoring approaches for pervious roads, sensing areas were set above and under the road; temperature and humidity sensors were established 50 cm above the ground (in the air) and 5–10 cm in the soil (underground) to serve as test points. The sensing positions were as described in Table 3 and Figure 4.

#### 2.3.4. Cloud Green Data System

**Figure 5:**
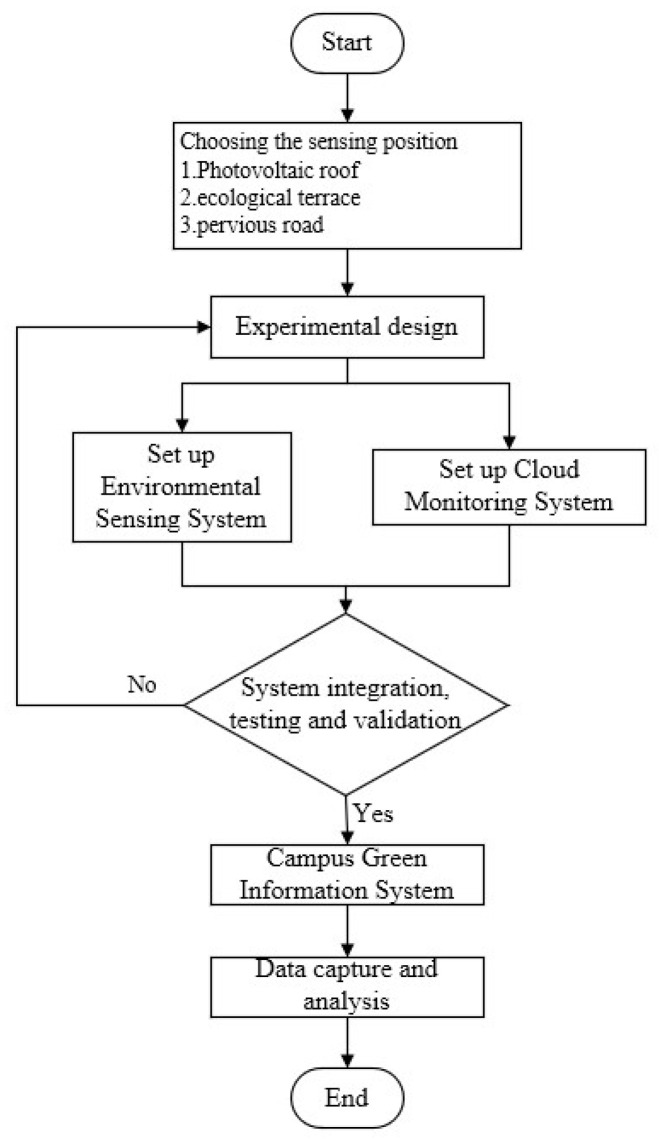
Structure of the cloud management monitoring system.

**Figure 6:**
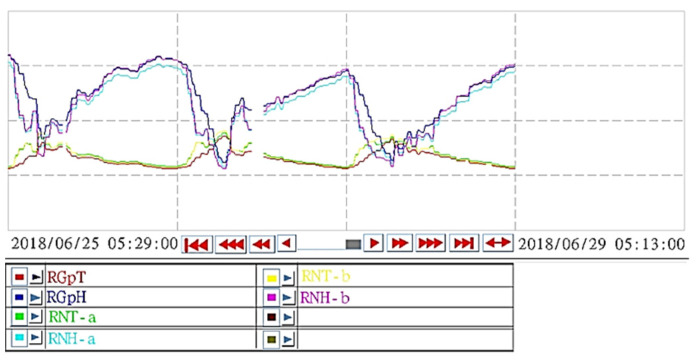
Historical data search in the campus green data monitoring system.

This study established a cloud monitoring system for the campus green information system. The system structure was as depicted in Figure 5. WebAccess graphic software was used to develop the cloud monitoring system, which processed, converted, and calculated the data gathered from sensors for instant display, graphical demonstration, and historical data search. The system was convenient and quick to learn for managers and could be operated through a browser. Instant data from the regional environment of each monitored area were gathered. Data were accompanied by photographs of the overall greening area to inform users of the status of the environment, sensing positions, and additional environmental greening benefits. The sensing results for each area were revealed in the information on campus green engineering benefits and quantified using the campus geographic map after the data were compared. The system was also designed with a historical data search page, which provided managers the ability to retrieve sensing data from previous time periods; the historical sensing data of up to 12 sensing points could be retrieved simultaneously (Figure 6). Sensor locations (i.e., the photovoltaic roof, ecological terrace, and pervious road) were encoded (Table 4). 

## 3. Environmental Data Analyses of the Green Information System

### 3.1. Benefit Analyses of the Photovoltaic Roof

This study monitored ambient temperature and humidity at the fixed photovoltaic system. Monitoring data were collected every 5 or 15 min over 5 consecutive days in March, April, and May to inspect the hourly mean temperature and humidity in higher (12:00–13:00) and lower (04:00–05:00) temperature periods. 

A photovoltaic roof has both a power generation function and a shading effect on a building. The measurement results for the influence of solar panels on the cooling of the temperature in the building indicate that the solar panels reduced building temperature and were beneficial regarding energy saving and human comfort. The shading effect of the solar panels during spring in high (12:00–13:00) and low (04:00–05:00) temperature periods were analyzed separately.

As Table 5 suggests, in low-temperature periods during 17–21 March, the ambient temperature on the roof under the panels was higher than that of the unshaded roof, and the air temperature without shade was higher than the air temperature under the panels. During the high-temperature periods, the roof and air temperatures under the solar panels were generally lower than those in unshaded areas. During 4–8 April, in the low-temperature period, the ground and air temperatures under the solar panels were generally lower than those in the unshaded area; during the high-temperature period, the ground and air temperatures under the panels were both lower than those in the unshaded areas. During 25–29 May, in the low-temperature period, the ground and air temperatures under the panels were lower than those in the unshaded areas. The highest temperature in May occurred at noon on 27 May and was the highest May temperature recorded since the establishment of the Taipei Weather Station. As displayed in Table 5, a comparison of the ambient temperatures on the roof and under the panels suggests that, in the high-temperature period (12:00–13:00), ground and air temperatures under the solar panels were lower than those in the unshaded areas. 

### 3.2. Benefit Analyses of the Ecological Terrace

#### 3.2.1. Environmental Greening Benefits of the Ecological Terrace (Green Roof) for the Top Floor

This study monitored the ambient temperature and humidity of the ecological terrace (green roof) and a top-floor classroom beneath the terrace. The environment was monitored from the middle of May (spring) to June (summer), and temperature and humidity data were gathered every 5 min for 8 consecutive days. The mean hourly temperature and humidity in the highest (12:00–13:00) and lowest (04:00–05:00) temperature periods were examined. The highest and lowest daily temperature periods were determined based on the mean hourly temperature in March. 

**Figure 7:**
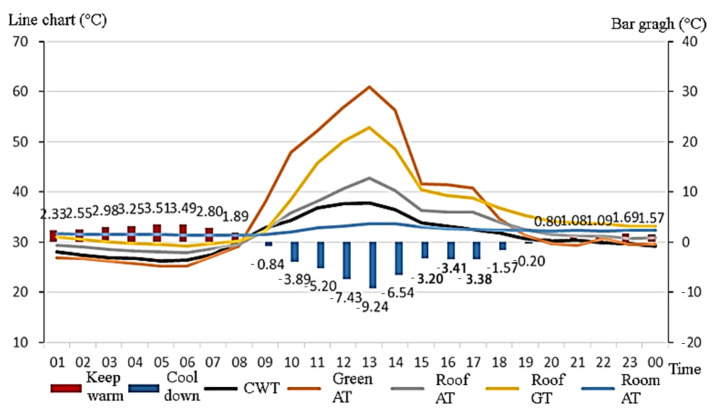
Ambient temperature trends of the ecological terrace on 27 May.

**Figure 8:**
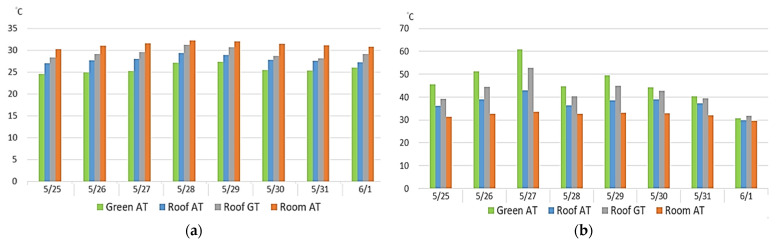
Environmental effects of the ecological terrace during (**a**) 04:00–05:00 and (**b**) 12:00–13:00.

The temperature measured at noon on 27 May was 38.2 °C, the highest temperature ever recorded in Taipei in May. The temperature was similar to summer temperatures in Taiwan; therefore, such data could be adopted as a reference for the benefits of the environmental cooling effect of green roofs in summer. This study collected mean hourly temperature data on 27 May (Figure 7) and integrated them with those provided by the Central Weather Bureau (code: CWT) for comparison. The roof temperature was slightly higher than the one measured by the Bureau because of the direct sunlight on the roof, but the trends in the air temperature data on the roof and those provided by the Bureau were similar. As Figure 8 reveals, the ambient temperature began to rise between 8:00 and 9:00 in the morning, and the temperature was highest between 12:00 and 13:00, when the ecological terrace had the maximum cooling effect (a temperature drop of 9.24 °C) on the space beneath. The ambient temperature decreased to approximately 30 °C between 20:00 and 21:00, and the ecological terrace began to exhibit an insulation effect on the space beneath. 

Table 6 and Table 7 present comparisons of the temperatures measured at all test points in the ecological terrace from 25 May to 1 June. The results indicate that, during the lower temperature period, the ecological terrace had the lowest temperature, followed by the air temperature on the roof. The highest temperature was that of the classroom, followed by the temperature on the roof itself, as displayed in Figure 8a. During the higher temperature period, the highest temperature was measured in the overall ecological terrace (green roof) environment, followed by the temperature on the roof itself; the lowest temperature occurred in the classroom, followed by the air temperature on the roof, as can be observed in Figure 8b. 

#### 3.2.2. Shading Effect of Ecological Terrace (Green Roof) on the Building

Green roofs possess advantages, including beautifying cities, cooling the top floors of buildings, and reducing heat island effects. This study analyzed the shading effect of an undermaintained ecological terrace on the top floor of a building during higher (12:00–13:00) and lower (04:00–05:00) temperature periods. 

**Figure 9:**
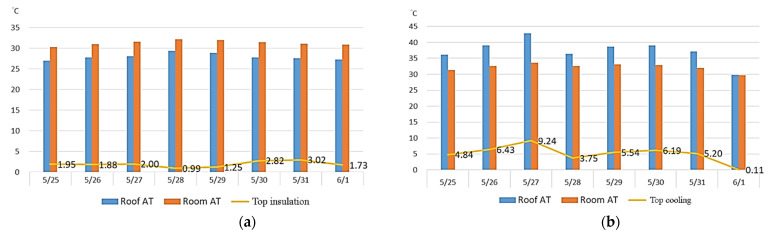
Comparison of the shading effect of the ecological terrace on the building during (**a**) 04:00–05:00 and (**b**) 12:00–13:00.

From 25 May to 1 June, the ambient temperatures on the roof and in the classroom beneath demonstrated that, in the lower temperature period, the air temperature in the classroom was higher than that of the green roof (Figure 9a). On 27 May, during the higher temperature period, the air temperature in the classroom was lower than the air temperature on the green roof (Figure 9b).

### 3.3. Benefit Analyses of the Cooling Effect of Pervious Roads

During the monitoring period, ambient temperature and humidity, as well as the temperature and humidity on and under the ground, on a campus pervious road were measured and the data were collected every 15 min. This study conducted the examination from January to March, with relatively stable monitored and transmitted data for analyses. The ambient temperature and humidity data for consecutive days were gathered, and the mean hourly temperature and humidity in the highest (12:00–13:00) and lowest (04:00–05:00) temperature periods were analyzed (Table 8). The daily highest and lowest temperature periods were determined based on mean hourly temperatures in March. 

**Figure 10:**
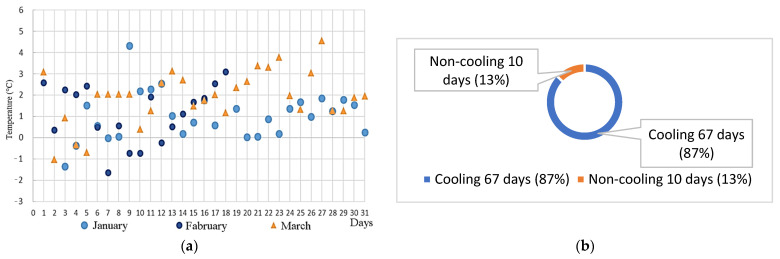
(**a**) Temperature distribution and (**b**) ratio of cooling days on the pervious road during 4:00–5:00 from January to March.

**Figure 11:**
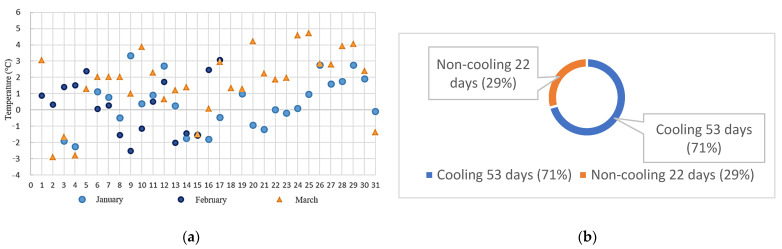
(**a**) Temperature distribution and (**b**) ratio of cooling days on the pervious road during 12:00–13:00 from January to March.

The ambient temperature on the road was compared with the temperature under the road. In Figure 10a, positive values refer to situations when the air temperature was lower than the soil temperature, and negative values signify the opposite. As Figure 10b indicates, during the lower temperature period, the ambient temperatures of 87% of the days from January to March were lower than the soil temperature underground. As Figure 11a discloses, during the higher temperature period, the ambient temperatures of 71% of the days from January to March were lower than the soil temperature. 

## 4. Results and Discussion

**Figure 12:**
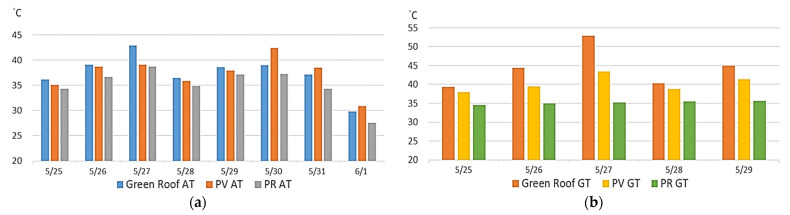
Comparison of overall environments: (**a**) air temperature (**b**) ground temperature.

The benefits of the photovoltaic roof, ecological terrace, and pervious road for environmental greening were compared using higher (12:00–13:00) and lower (4:00–5:00) temperature periods. As Figure 12 suggests, the environmental cooling benefit of the pervious road was strongest, based on the results of both ambient and ground temperatures. 

### 4.1. The Benefit of the Solar Photovoltaic Roof for Building Shading

(a) Roof shading benefits of solar panels

During the cold period from 4 to 5 in the morning, the air temperature under the solar panel was lower than the ambient air temperature on the top floor. When the ambient temperature at night was lower than 25 °C, the ground below the solar panel generally had a thermal insulation effect, and the maximum temperature was 2.14 °C; if it was higher than 25 °C, the ground below the solar panel generally had a cooling effect, and the maximum temperature drop was 3.7 °C.

During the hot period from 12 to 13 noon on the solar photovoltaic roof, the air temperature under the solar panel was generally lower than the ambient air temperature on the top floor, and the maximum temperature drop was 2 °C. When the ambient temperature during the day was higher than 25 °C, the ground temperature under the solar panel had a cooling effect, and the maximum temperature drop was 5.77 °C; if it was lower than 25 °C, the ground below the solar panel generally had a thermal insulation effect, and the maximum temperature was 2.4 °C.

According to Dominguez, Kleissl, and Luvall (2011), inclined solar panels are conducive to air flow below and do not easily collect heat, so the temperature of the air and the ground under an inclined panel is lower than that of the flat-mounted solar panel. The environmental benefits of this case are similar to their effects.

### 4.2. The Environmental Benefits of Green Roofs on the Top Floor and the Shading Effects of Green Roofs on Buildings

(a) The highest temperature day in May

Comparing the daily temperature data of the Central Weather Bureau with the campus environment monitoring system, it can be seen that the temperature trend monitored in this study was similar to the temperature data trend of the Central Meteorological Administration. They were all slightly higher than the temperature data of the Central Meteorological Bureau, and the climate temperature on that day was similar to summer temperatures in Taiwan, which can be used as the benefit of green roofs affecting the top floor environment in summer. From the statistical results, it is known that green roofs are built in environments with high ambient temperatures. It may be better for cooling the space under buildings.

(b) Shading effect of green roofs on buildings

Because it was not easy to monitor and compare the air temperature and humidity of the classrooms under the ungreen roof, the roof air temperature and humidity data were used for comparison. The statistical results show that the green roof contained a soil layer during the colder period from 4 to 5 in the morning, which had a thermal insulation effect on the space below, and the thermal insulation effect was about 1~3 °C. The average temperature was 1.95 °C, and the green roof received most of the heat during the hot period from 12:00 to 13:00, which had a cooling effect on the space below. The cooling effect was about 0.5~9 °C, and the average temperature drop was 5.16 °C.

### 4.3. Comparison of the Greening Benefits of the Campus Environment and the Ratio of the Number of Days That the Permeable Pavement Soil Absorbs Heat

(a) Comparison of campus greening benefits

The data was collected for a total of five days. It can be clearly seen that the benefits of the greening sites regarding the cooling of the ambient air and the ground are in the order of permeable pavement > solar photovoltaic roof > ecological terrace (green roof). It may be because the surrounding environment of the permeable pavement was open and planted with flowers, plants, and trees to increase the cooling effect of the surrounding environment, and the cooling part of the ground may be because the sensor was buried about 5~10 cm below the soil of the permeable pavement, so the cooling effect better.

(b) Ratio of days in which pervious pavement soil absorbed heat

A permeable pavement made of grass-planted bricks on campus has been monitored for about a year since last year. Due to the testing of communication and power supply in the first half of the year, the data collection was incomplete and was not included in the comparison. This analysis compared the soil air temperature under the permeable pavement and the above-ground air temperature. The results show that the cooling days during the colder period from 4 to 5 a.m. in the morning from January to March accounted for 89% of the cooling days compared with 71% in the warmer period from 12 to 13 a.m. The reason was that the ambient temperature in the daytime was too high and the cooling effect was not significant. From January to March, the maximum cooling effect of the hours during the day was 4.75 °C, the average maximum temperature drop in the month was 2.32 °C, and the maximum cooling effect was 4.33° from 4 to 5 in the morning. The monthly average maximum temperature drop reached 2.22 °C.

## 5. Conclusions

This study established a wireless environmental monitoring management system based on IoT technology and the smart city concept to monitor and quantify the long-term benefits of green engineering on campus environments. Three greening engineering areas, namely a photovoltaic roof, an ecological terrace (green roof), and a pervious road (grass pavers), were analyzed. The results may be summarized as follows.

1. Feasibility of the wireless environmental monitoring management system: This study built a wireless environmental sensing management system for the greening environment on a campus and successfully monitored physical quantities in the green engineering environments. These quantities could be applied in other green engineering areas and the sensing data could be uploaded to a cloud system for data management. By using a visual monitoring interface to display the environmental monitoring situations, managers could develop system functions such as instant data display, historical trend figure establishment, and report queries and outputs according to their needs. This study can be an index for Taiwan’s EEWH.
2. Shading effect of the photovoltaic roof during spring (March–May): The air temperatures under the fixed solar panels were generally lower than the ambient temperature whether during 4:00–5:00 or 12:00–13:00, and the roof temperature under the panels was affected by the ambient temperature. When the ambient temperature was below 25 °C, the solar panels had an insulation effect on the roof of the building during both 4:00–5:00 and 12:00–13:00, with an optimal insulation effect of 2.45 °C. When the ambient temperature was above 25 °C, the panels had a cooling effect on the roof of the building whether during 4:00–5:00 or 12:00–13:00, with an optimal cooling effect of 5.77 °C.
3. Shading effect of the ecological terrace (green roof) on the building: During the lower temperature period (4:00–5:00), the ecological terrace had an insulation effect on the space beneath, with an effect of approximately 1–3 °C and a mean insulation of 1.95 °C. During the higher temperature period (12:00–13:00), it presented a cooling effect on the space beneath, with an effect of approximately 0.5–9 °C and a mean cooling temperature of 5.16 °C. The green roof cooled and insulated the space beneath during the day and at night, respectively.
4. Environmental cooling effect of the pervious road: The cooling effect of the three greening areas on the air and ground temperatures decreased in the following order: pervious road > photovoltaic roof > ecological terrace. The pervious road presented the optimal cooling effect of the three. During a higher temperature period (12:00–13:00), the pervious road had a maximum cooling temperature of 4.75 °C, with a monthly mean minimum cooling temperature of 2.32 °C. During a lower temperature period (4:00–5:00), it had a maximum cooling temperature of 4.33 °C and a monthly mean cooling temperature of 2.22 °C.

## Figures and Tables

**Table 1:** Sensing positions on the photovoltaic roof.

Site	Photovoltaic Roof			
**Sensing Area**	**Shaded Area**		**Unshaded Area**	
Test point positions	In the air	On the roof	In the air	On the roof

**Table 2:** Sensing positions on the ecological terrace.

Site	Ecological Terrace			
**Sensing Area**	**Greening Area**		**Unshaded Area**	
Test point positions	In the air	On the roof	In the air	On the roof

**Table 3:** Sensing positions on the pervious road.

Site	Pervious Road (Grass Pavers)	
**Sensing Area**	**Pervious Road**	
Test point positions	Above ground (in the air)	In the soil (underground)

**Table 4:** Sensor location codes.

Items	Codes
Air	A
Ground	G
Underground	UG
Temperature	T
Humidity	H
Difference	D
Photovoltaic roof	PV
Ecological terrace (green roof)	Green
Pervious road	PR
Roof	Roof
Room	Room

**Table 5:** Shading effect of the photovoltaic panels.

Date	Period	PV AT (°C)	PV GT (°C)	Roof AT (°C)	Roof GT (°C)	D AT (°C)	D GT (°C)
3/17	Low	19.22	19.84	19.96	19.56	0.74	−0.28
	High	22.14	22.38	23.4	22.89	1.26	0.5
3/18	Low	20.27	20.89	21.04	20.63	0.77	−0.26
	High	32.23	28.95	32.2	32.34	−0.03	3.39
3/19	Low	21.97	22.71	22.6	22.31	0.64	−0.4
	High	33.11	30.95	32.85	34.87	−0.26	3.92
3/20	Low	23.04	23.94	23.76	23.81	0.72	−0.13
	High	20.41	21.31	21.1	18.91	0.69	−2.4
3/21	Low	16.49	17.42	16.88	15.28	0.39	−2.14
	High	17.04	17.77	17.85	17.7	0.8	−0.08
4/4	Low	22.44	32.59	23.1	35.86	0.66	3.26
	High	35.08	32.59	37.08	35.86	1.99	3.26
4/5	Low	23.91	31.06	24.49	34.76	0.58	3.7
	High	31.22	31.06	32.1	34.76	0.88	3.7
4/6	Low	24.22	22.34	24.95	21.5	0.72	−0.85
	High	20.27	22.34	21.08	21.5	0.81	−0.85
4/7	Low	15.92	18.73	16.56	19.21	0.65	0.47
	High	19.21	18.73	20.92	19.21	1.7	0.47
4/8	Low	15.7	25.86	16.44	28.16	0.74	2.3
	High	27.93	25.86	28.7	28.16	0.77	2.3
5/25	Low	26.52	27.77	27.34	28.06	0.82	0.29
	High	33.81	33.88	35.02	37.89	1.21	4.01
5/26	Low	27.9	29.21	28.74	29.61	0.84	0.4
	High	37.51	35.96	38.72	39.45	1.21	3.49
5/27	Low	27.57	29.09	28.43	29.81	0.86	0.73
	High	39.16	37.66	39.04	43.43	−0.12	5.77
5/28	Low	29.14	30.37	30.04	31.06	0.9	0.69
	High	34.84	34.41	35.86	38.73	1.02	4.31
5/29	Low	28.76	29.91	29.64	30.36	0.88	0.44

**Table 6:** Ambient temperatures of the ecological terrace during 04:00–05:00.

Date	Roof AT (°C)	Room AT (°C)	Top Cooling Effect (°C)
5/25	26.99	30.27	1.95
5/26	27.72	31.01	1.88
5/27	28.03	31.53	2.00
5/28	29.36	32.19	0.99
5/29	28.90	31.96	1.25
5/30	27.78	31.49	2.82
5/31	27.56	31.11	3.02
6/01	27.21	30.81	1.73

**Table 7:** Ambient temperatures of the ecological terrace during 12:00–13:00.

Date	Roof AT (°C)	Room AT (°C)	Top Cooling Effect (°C)
5/25	36.17	31.33	4.84
5/26	39.04	32.61	6.43
5/27	42.86	33.62	9.24
5/28	36.40	32.65	3.75
5/29	38.61	33.07	5.54
5/30	39.01	32.82	6.19
5/31	37.15	31.95	5.20
6/01	29.78	29.67	0.11

**Table 8:** Analysis period of pervious road data.

Month	January	February	March
Number of days	31 days	28 days	31 days
Data collection rate in the low-temperature period	90%	64%	100%
Data collection rate in the high-temperature period	87%	61%	100%

## Data Availability

The data presented in this study are available within the article.
